# Synergistic combination of ceftazidime and avibactam with Aztreonam against MDR *Klebsiella pneumoniae* in ICU patients

**DOI:** 10.1038/s41598-025-88965-7

**Published:** 2025-02-11

**Authors:** Sally Khattab, Aya Mohamed Askar, Hidi A. A. Abdellatif, Amira A. A. Othman, Amal H. Rayan, Hasnaa Azab

**Affiliations:** 1 Microbiology and Immunology Department, Faculty of Medicine, Suez Canal University, Ismailia, Egypt; 2 Clinical and Chemical Pathology Department, Faculty of Medicine, Suez Canal University, Ismailia, Egypt; 3 Medical Biochemistry and Molecular Biology Department, Faculty of Medicine, Suez Canal University , Ismailia, Egypt; 4 Oncology Diagnostic Unit, Faculty of Medicine, Suez Canal University, Ismailia, Egypt; 5 Medical Biochemistry and Molecular Biology Department, Faculty of Medicine, King Salman International University, Tur Sinai, South Sinai Egypt; 6 Internal Medicine Department, Faculty of Medicine, Suez University, Suez, Egypt; 7 Department of Basic Medical Science, College of Medicine, AlMaarefa University, Riyadh, Kingdom of Saudi Arabia; 8 Faculty of Medicine, Suez Canal University, Ismailia, Egypt

**Keywords:** *Klebsiella pneumoniae*, Synergy, mCIM, eCIM, E-test, Carbapenemase, Immunology, Microbiology

## Abstract

The proliferation of multidrug-resistant, metallo-beta-lactamase-producing *Klebsiella pneumoniae* (MBL-producing *K. pneumoniae*) poses a major threat to public health resulting in increasing treatment costs, prolonged hospitalization, and mortality rate. Treating such bacteria presents substantial hurdles for clinicians. The combination of Aztreonam (ATM) and ceftazidime/avibactam (CAZ/AVI) is likely the most successful approach. The study evaluated the in vitro activity of CAZ/AVI in combination with ATM against MBL-producing *K. pneumoniae* clinical isolates collected from Suez Canal University Hospital patients. Carbapenem-resistant *K. pneumoniae* were isolated and identified from different specimens. The presence of metallo-β-lactamases was detected phenotypically by modified carbapenem inactivation method (mCIM) and EDTA-CIM (eCIM) testing, and genotypically for the three metallo-β-lactamase genes: *bla*NDM, *bla*IMP, and *bla*VIM by conventional PCR method. The synergistic effect of CAZ/AVI with ATM against MBL-producing *K. pneumoniae* was detected by ceftazidime-avibactam combination disks and E-test for antimicrobial susceptibility testing. Out of the 65 K. pneumoniae isolates recovered, 60% (39/65) were carbapenem-resistant (CRKP). According to the mCIM and eCIM tests, 89.7% (35/39) of CRKP isolates were carbapenemase-positive, and 68.6% (24/35) were metallo-β-lactamase (MBL)-positive. By using the conventional PCR, at least one of the MBL genes was present in each metallo-bata-lactamase-producing isolate: 8.3% carried the *bla*VIM gene, 66.7% the *bla*NDM, and 91.7% the *bla*IMP gene. After doing the disk combination method for ceftazidime-avibactam plus Aztreonam, 62.5% of the isolates shifted from resistance to sensitivity. Also, ceftazidime/avibactam plus Aztreonam resistance was reduced markedly among CRKP using the E-test. The addition of Aztreonam to ceftazidime/avibactam is an effective therapeutic option against MBL-producing *K. pneumoniae*.

**Clinical Trials Registry**: Pan African Clinical Trials Registry. Trial No.: PACTR202410744344899. Trial URL: https://pactr.samrc.ac.za/TrialDisplay.aspx?TrialID=32000

## Introduction

In recent years, the spread of carbapenem-resistant *Klebsiella pneumoniae*, particularly strains producing metallo-β-lactamase, has become a challenge of particular importance in clinical practice worldwide. MBL-positive CRKP exhibits resistance to nearly all beta-lactam antibiotics, including carbapenems, leaving very limited therapeutic options^1^. Several recent studies have pointed out the role of the *bla*NDM, *bla*IMP, and *bla*VIM genes in determining this resistance, with *bla*NDM being the most widespread in many countries^2^. Ceftazidime/Avibactam in combination with Aztreonam has been an effective combination against MBL-mediated resistance since Aztreonam is stable against MBLs and Ceftazidime/Avibactam inhibits serine beta-lactamases^3^. The efficacy of this combination against MBL producers among CRKP in our region remains underexplored.

The global rise of multidrug-resistant (MDR) bacteria has heightened concerns about infections that are untreatable with conventional antibiotics. Carbapenem-resistant *Klebsiella pneumoniae* (CRKP), in particular, poses a serious public health threat, increasing mortality rates among critically ill patients and exacerbating the financial burden of hospital stays worldwide. According to the US Centers for Disease Control and Prevention, the infection rate of carbapenem-resistant Enterobacterales (CRE) is 57/100,000, with in-hospital mortality rates of 33.50% for nosocomial infections and 43.10% for bloodstream infections^4^. Notably, approximately 50% of patients with *K. pneumoniae* pneumonia succumb to the infection^5^. In Egypt, CRKP prevalence ranges from 31.3% in university hospitals^6^ to 25.4% in tertiary care hospitals^7^, while in Greece, the rate reaches 66.3%^8^.

One or a combination of the following mechanisms mediate resistance to carbapenems: (1) the carbapenemases production which hydrolyzes carbapenems, such as the serine β-lactamases *Klebsiella pneumoniae* carbapenemase (KPC) (Ambler class A), metallo-β-lactamase (MBL) including New Delhi MBL (NDM) or Verona integron-encoded MBL (VIM), imipenemase (IMP) (Ambler class B) and OXA-48-like carbapenemases (Ambler class D), and less frequently, the production of Extended-spectrum β-lactamases (ESBLs) and/or Ambler class C β-lactamase (AmpC) enzymes; (2) reduced permeability as a result of defective expression of specific outer membrane proteins; (3) drug efflux across the outer membrane; and (4) the production of a modified or low-affinity target, which is more significant in Gram-positive bacteria^9^. MBLs, out of all the carbapenemases, are currently the more concerning because of their ability to hydrolyze all β-lactams and carbapenems except Aztreonam (monobactam), and they are not inhibited by boronic acid inhibitors (vaborbactam), novel diazabicyclooctane (DBO) β-lactamases (avibactam, relebactam), or classical serine β-lactamase inhibitors (e.g., clavulanic acid, tazobactam, sulbactam)^10^.

Aztreonam has been the only monocyclic lactam antibiotic that is currently accessible for clinical use and effective against MBLs since it was approved by the US Food and Drug Administration (FDA) in 1986^11^. Unfortunately, most MBL-Enterobacterales also co-harbor additional ESBL or AmpC β-lactamase genes, which makes monobactam ineffective against MBL producers and imparts Aztreonam resistance^12^. Ceftazidime/avibactam is a combination of beta-lactamase inhibitor (avibactam) and cephalosporin (ceftazidime). Avibactam inhibits serine carbapenemases such as *Klebsiella pneumoniae* carbapenemase (KPC), AmpC beta lactamases, OXA-48, and ESBLS. By blocking class A, class C, and some class D beta-lactamases, avibactam restores ceftazidime’s action and overcomes resistance brought on by carbapenemases such as KPC. The combination, however, is ineffective against class B beta-lactamases, including VIM, NDM, and other metallo-beta lactamases^13^. Aztreonam is stable to the hydrolysis of MBLs like NDM, and avibactam protects Aztreonam from hydrolysis by serine β-lactamases, so the combination of Ceftazidime/avibactam plus Aztreonam is a valuable option to treat infections by beta-lactamase-producing Gram-negative pathogens producing serine β-lactamases, MBLs, or both^14^.

Currently, there are no commonly accepted antimicrobial susceptibility testing methods to assess the synergic effect of CAZ/AVI plus ATM. “Time-kill kinetics” and “checkerboard” assays are labor-intensive, complicated, and interpretation-challenging testing procedures of synergistic effectiveness. The “E-strip-disc diffusion”, “E-strip stacking”, and “E-strip cross” assays are somewhat subjective, not very labor-intensive, and accurate^15^.

This study aims to evaluate the in vitro activity of CAZ/AVI in combination with ATM against MBL-producing *K. pneumoniae* clinical isolates collected from patients of Suez Canal University Hospital by disc diffusion and E-test methods.

## Subject and methods

### Study population and design

This observational cross-sectional descriptive study was conducted at the Suez Canal University Hospitals in Ismailia, Egypt, from December 2022 to June 2024. Samples were collected from different intensive care units (ICUs) and other hospital wards and were processed in the Medical Microbiology and Immunology Department laboratory, Faculty of Medicine, Suez Canal University.

#### Ethical considerations

The current study was implemented in coordination with the guidelines of the Declaration of Helsinki. Ethical approval was gained from the Research Ethics Committee of the Faculty of Medicine, Suez Canal University, Egypt, # 5060. The patients provided written informed consent, which addressed all the steps of the study and their right to withdraw at any time. The minor patient’s informed consent was obtained from a parent, which addressed all the steps of the study and their right to withdraw at any time.

#### Inclusion criteria

Immunocompromised, debilitated patients, patients with serious underlying diseases of both sexes (male and female), and patients of all age groups who showed signs and symptoms of pyogenic infections and agreed to participate were included in this study. Samples included urine from catheterized and non-catheterized patients, respiratory specimens from intubated and non-intubated patients, Pus and wound swabs, pleural fluid, and blood.

#### Exclusion criteria

Patients who received antibiotic treatment in the last 48 h or refused to participate were excluded.

### Study procedures

#### Full history taking, and thorough physical examination

All enrolled patients were asked and examined for underlying medical conditions or certain types of healthcare exposures, such as:

- Immunocompromising conditions.
- Recent frequent or prolonged stays in health care settings.
- Invasive medical devices such as breathing tubes, feeding tubes, and central venous catheters.
- Open wounds.
- A history of taking certain antibiotics for long periods.

For every patient admitted to internal wards or intensive care units, the following data was found age, length of stay, number of days needed for mechanical ventilation, and survival until hospital discharge. Additionally, microbiological results were obtained to identify *K. pneumoniae-*positive cultures.

#### Samples collection and preservation

Various samples (urine, sputum, endotracheal aspirate, pleural fluid, pus, and wound exudate) were collected aseptically^16^ throughout the study for presumptive *K. pneumoniae* isolation and identification. The samples were immediately transported to the laboratory in sterile containers and stored at 4 °C for a maximum of 2 h before processing to maintain sample integrity and prevent bacterial overgrowth. A total of 365 clinical samples were analyzed for bacterial isolation.

#### Bacterial isolation and identification

For bacterial isolation, all collected samples were inoculated into blood agar and MacConkey agar media (Oxoid, UK) and incubated aerobically at 35 ± 2℃ for 24 h. For initial identification of the isolates, they underwent a hanging drop test, and biochemical tests including “catalase, citrate, oxidase, oxidative fermentation, indole, Methyl Red (MR), Voges-Proskauer (VP), urease, H2S production, and gelatin hydrolysis, pigment, and MUG test”.

#### Phenotypic detection of metallo-β-lactamases by modified carbapenem inactivation method (mCIM) and EDTA-CIM (eCIM) testing

The Clinical and Laboratory Standards Institute (CLSI) adopted “modified carbapenem inactivation method” (mCIM) and “EDTA-modified carbapenem inactivation method” (eCIM) as a combination phenotypic test to detect and differentiate between serine and metallo-based carbapenemases for Enterobacterales^17^.

One “microliter loopful of the test isolate” was suspended in 2 ml of “trypticase soy broth” (TSB), and a “meropenem disc” was added to the suspension. The culture was incubated for 4 h at 35 °C. The control strain of *K. pneumoniae* MGH 78,578 (a carbapenem-sensitive strain) was adjusted in sterile saline with 0.5 “McFarland standard and streaked” in three directions on “Mueller–Hinton Agar” (MHA) plates to ensure an even and uniform cell lawn. After transferring the disc from the TSB suspension onto an MHA plate, the plate was left to incubate for the full night at 35 °C. To achieve a “final EDTA concentration” of 5 mM for every isolate, 20 µL of the 0.5 M EDTA was filled into a second 2-mL TSB tube intended for the eCIM test, which was labeled. Then the same steps as in mCIM were performed. The mCIM and eCIM tubes were processed in parallel. The “meropenem discs” from the mCIM and eCIM tubes were placed on the same MHA plate inoculated with the meropenem-susceptible *K. pneumoniae* MGH 78,578 indicator strain. Following incubation, the diameter of the inhibition zone was measured.

##### For the mCIM test

Carbapenemase positive test strain: if it showed an inhibition zone 6–15 mm or the presence of pinpoint colonies within a 16–18 mm zone. Carbapenemase negative test strain: if it showed inhibition zone ≥ 19 mm. Intermediate test strain: if it showed inhibition zone 16-18 mm.

##### For the eCIM test

When the mCIM test showed positive results; metallo-β-lactamase positive strain: a zone diameter increases of ≥ 5 mm for eCIM in comparison to mCIM. Metallo-β-lactamase negative strain: A ≤ 4-mm increase in zone diameter for eCIM compared to mCIM.

#### Molecular detection of metallo-β-lactamase genes by conventional PCR

Bacterial DNA was extracted using the “QIAprep Miniprep DNA Purification Kit” (“QIAGEN, Germany”) according to the manufacturer’s instructions for the detection of *bla*NDM*, bla*IMP*, and bla*VIM with a set of primers^18^ as described in Table 1. The reaction mixture was prepared in a total volume of 25 μl including:

- 5 μl of template DNA,
- 12.5 μl of 2X ABT Red master mix (Applied Biotechnology Co. Ltd, Egypt), and
- 2 μl of both forward and reverse primers
- the volume was then completed with sterile distilled water up to 25 μl.

**Table 1:** Gene-specific primers used to detect *metallo-β-lactamase genes* in *K. pneumoniae* isolates.

Gene targeted	Primers (5′ → 3')	Product size(bp)
*bla*NDM	**F-**ACCGCCTGGACCGATGACCA **R**-GCCAAAGTTGGGCGCGGTTG	236
*bla*IMP	**F**-GAAGGCGTTTATGTTCATAC **R**-GTATGTTTCAAGAGTGATGC	587
*bla*VIM	**F**-GTTTGGTCGCATATCGCAAC **R**-AATGCGCAGCACCAGGATAG	382

Reaction mixtures without a DNA template served as negative controls. Amplification was carried out in a thermal cycler (Peltier Thermal cycler, MJ Research, U.S.A). PCR cycling conditions included “initial denaturation” at 94 °C for 3 min, 35 cycles of 94 °C for 1 min, 56–58 °C for 1 min, 72 °C for 1 min for extension, and a final polymerization at 72 °C for 10 min”. Amplified fragments were separated by electrophoresis in 2% agarose gel at 5 V/cm for 2 h and observed by a UV transilluminator.

#### Antimicrobial sensitivity testing

##### Standard disc diffusion method

Phenotypic detection of antibiotic resistance was done using the “Kirby-Bauer disk diffusion method” on Mueller Hinton agar (Oxoid, UK) and incubated at 35 °C for 16–18 h according to Clinical and Laboratory Standard Institute guidelines of CLSI, 2022, control strain of *K. pneumoniae* MGH 78,578 was used^15^. The antibiotics used: Ampicillin (10 µg), Amoxicillin-clavulanate (20/10 µg), Cefepime (30 µg), Ceftazidime (30 µg), Cefotaxime (30 µg), Imipenem (10 µg), Meropenem (10 µg), Gentamicin (10 µg), Amikacin (30 µg), Ciprofloxacin (5 µg), Levofloxacin (5 µg). All antibiotics were purchased from “Sigma Chemicals (St. Louis, USA)”.

##### Ceftazidime/avibactam combination method with Aztreonam discs

The test organism was first added to the MHA plates through inoculation. Following that, 30 µg/20 µg of ceftazidime/avibactam antibiotic discs were placed on them, and they were incubated for 1 h at 35 ± 2 °C. After this incubation, the ceftazidime/avibactam disks were removed, and the Aztreonam discs were placed in the same location and incubated for 1 h at 35 ± 2 °C. The 1-h duration was sufficient for antibiotic diffusion into the medium, as confirmed by the observed zones of inhibition. The following day, a zone of inhibition was noted following CLSI 2024 guidelines for disc diffusion testing, synergy was considered present if the zone diameter of the replacement AZT disc was ≥ 21 mm^19^.

##### MIC of ceftazidime/avibactam alone and in combination with Aztreonam

For the minimal inhibitory concentration (MIC) determination, ceftazidime/avibactam and Aztreonam E-test strips were utilized. After an hour of incubation, one of the two ceftazidime/avibactam E-test strips was removed, and the imprint of the removed E-test strip was covered with an Aztreonam E strip. The plates underwent another 18-h incubation period at 35 ± 2 °C.

### Statistical analysis

Data was coded and entered into the computer statistical program. All statistical analyses were performed using the Statistical Package for Social Science (SPSS) version 25 (Inc, Chicago, Illinois, USA). Data presentation was performed via tables and graphs. Qualitative data were presented as numbers and percentages while quantitative data were presented as mean ± Standard Deviation. Fisher’s exact tests were used for qualitative variables. ANOVA test was used to compare the Zone diameter between groups and Tukey Post Hoc test was used to assess the significance between each two groups. A *p-value* of < 0.05 was considered statistically significant.

## Results

### Isolation, identification, and phenotypic detection of *K. pneumoniae* isolates

A total of 365 examined clinical samples were analyzed for bacterial isolation. A total of 65 K*. pneumoniae* isolates were recovered. 60% (39/65) of the isolates were CRKP identified by either being meropenem and/or imipenem resistant.

The patients’ ages ranged from 16 to 75, with a mean of 44.6 and a standard deviation of ± 20.4. The CRKP isolates were obtained more frequently from males (61.5%) than females (38.5%). The highest rate of CRKP isolation was from endotracheal aspirates (46.7%), while the lowest rate was from sputum and blood (10%) from ICUs of Suez Canal University hospitals (Table 2).

**Table 2:** Demographic and sample distribution of patients with carbapenem-resistant K. pneumoniae (N = 39).

Category	Value
Gender N (%)	
Male	24 (61.5%)
Female	15 (38.5%)
Participants age	
Range	16 – 75 years
Mean ± SD	44.6 ± 20.4
Type of specimen N (%)	
Endotracheal aspirates	18 (46.7%)
Urine	8 (20%)
Pus	5 (13.3%)
Sputum	4 (10%)
Blood	4 (10%)
Total	39 (100%)

Preliminary isolation and identification of *K. pneumoniae* were based on Gram-negative staining and colony morphology. The isolates were Gram-negative, non-motile bacilli, forming small (3–5 mm), gray, moist, and often mucoid colonies on blood agar. On MacConkey agar, the colonies appeared pink-yellow due to lactose fermentation. Biochemical tests confirmed the identification of *K. pneumoniae*, with positive results for catalase, citrate, VP, urease, and MUG tests, and negative results for oxidase, indole, MR, H_2_S production, gelatin hydrolysis, and pigment production. The isolates were also fermentative in oxidative fermentation tests.

### Phenotypic detection of metallo-β-lactamases by modified carbapenem inactivation method (mCIM) and EDTA-CIM (eCIM) testing

**Fig. 1:**
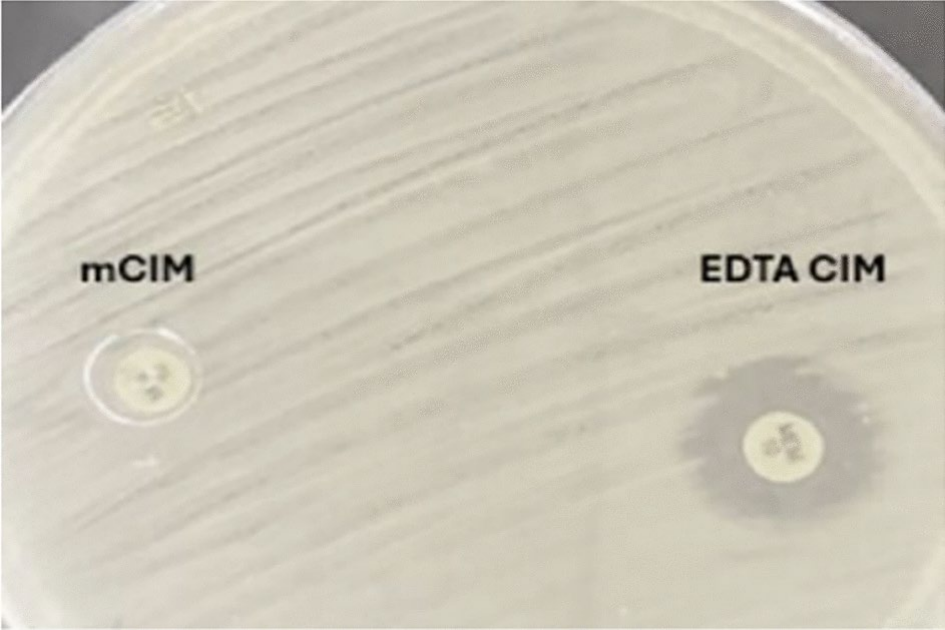
mCIM and eCIM testing showed an increase of zone diameter ≥ 5 mm after adding EDTA and they were considered as metallo-β-lactamase positive isolates.

Modified carbapenem inactivation method (mCIM) and EDTA-CIM (eCIM) tests resulted in 35 (89.7%) CRKP isolates showed a zone diameter of 6–15 mm for meropenem disc and interpreted as carbapenemase positive, while 1 (2.6%) isolate showed a 17 mm zone diameter that was interpreted as carbapenemase intermediate and 3 (7.7%) isolates showed zone diameters of ≥ 19 mm and interpreted as carbapenemase negative (Table 3). A total of 24 isolates (68.6%) showed an increase of zone diameter ≥ 5 mm after adding EDTA and they were considered Metallo-β-lactamase positive isolates (Table 4, Fig. 1).

**Table 3:** Modified carbapenem inactivation method results for the 39 CRKP isolates.

CRKP isolates	Number of specimens (N = 39)	Percentage (%)
Positive	35	89.7%
Intermediate	1	2.6%
Negative	3	7.7%

**Table 4:** EDTA-CIM testing for 35 carbapenemase-positive CRKP isolates.

	Number of specimens (N = 35)	Percentage
Positive	24	68.6%
Negative	11	31.4%

### Molecular detection of metallo-β-lactamase genes by conventional PCR

**Fig. 2:**
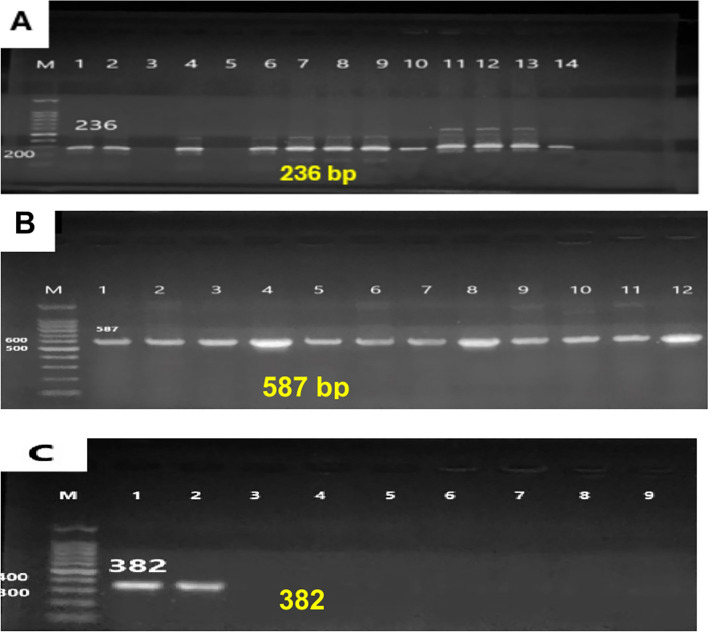
Detection of metallo-beta lactamase genes by agarose gel electrophoresis. Lane M shows a 100 bp molecular strand DNA ladder, (**A**) *bla*NDM *gene*; All Lanes show positive specimens (236 bp), except lane (3, 5), (**B**) *bla*IMP *gene*; All Lanes show positive specimens (587 bp), (**C**) *bla*VIM *gene*; 2 Lanes show positive specimens (382 bp).

By conventional PCR, molecular testing was done to detect the 3 MBL genes in the 24 Metallo-β-lactamase positive isolates. All 24 isolates harbored at least one of the MBL genes (*bla*IMP, *bla*NDM_*,*_ and *bla*VIM). A total of 22 isolates (91.7%) harbored *bla*IMP gene, 16 isolates (66.7%) harbored *bla*_*NDM*_ and 2 isolates (8.3%) harbored *bla*_*VIM*_ (Table 4, Fig. 2A–C). Full-length gel images with membrane edges are included in the Supplementary Information file. The gels were not physically cut before imaging.

### Antibiotic susceptibility test

#### Standard disk diffusion method

**Fig. 3:**
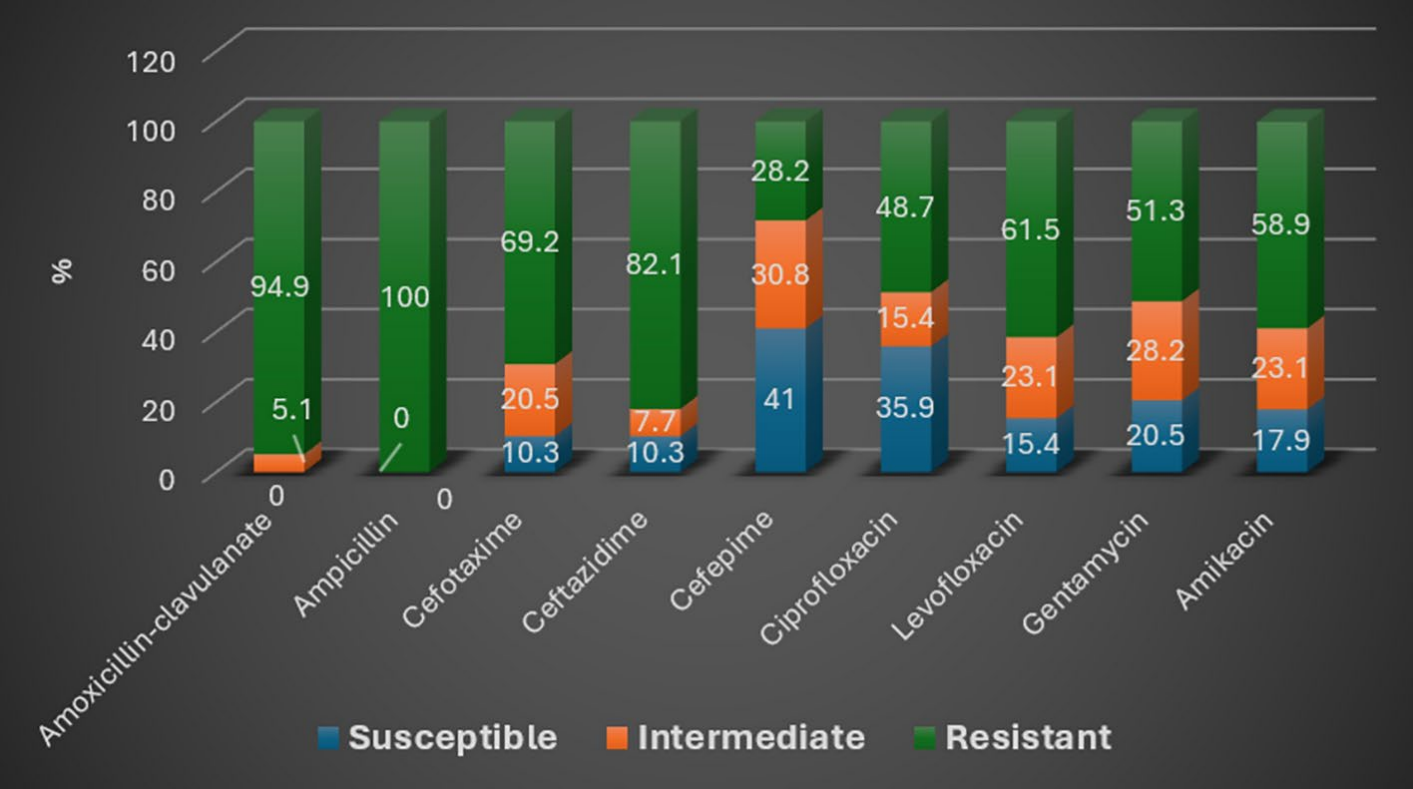
Antibiotic susceptibility pattern of the 39 CRKP isolates by Kirby-Bauer disk diffusion method.

Antibiotic susceptibility testing was done on the 39 *K. pneumoniae* isolates by the Kirby-Bauer disk diffusion method. The highest resistance was for Ampicillin, Amoxicillin-clavulanate, and Ceftazidime antibiotics as they showed 100%, 94.9%, and 82.1% resistance respectively. In contrast, the least resistance was for Cefepime 28.2% (Table 6, Fig. 3).

#### Ceftazidime/avibactam susceptibility testing alone and in combination with aztreonam (disc replacement method)

None of the isolates showed zone diameter ≥ 21 mm when testing Aztreonam disc susceptibility by the Kirby-Bauer method, while 2 isolates (8.3%) were ≥ 21 mm for Ceftazidime-Avibactam disc. However, after doing the combination method for Ceftazidime-Avibactam plus Aztreonam, synergy was observed in 15/24 (62.5%) of the resistant isolates, inhibition zone became ≥ 21 mm (Tables 7 and 8).

None of the sensitive isolates carried the *bla*VIM gene. None of the isolates showed zone diameters ≥ 21 mm for Aztreonam alone, while 2 isolates (8.3%) were sensitive to Ceftazidime/Avibactam alone. However, the combination of Ceftazidime/Avibactam + Aztreonam resulted in 15 isolates (62.5%) becoming sensitive, demonstrating a significant synergistic effect (p < 0.001). Among the 15 isolates that became sensitive to the combination of Ceftazidime-Avibactam + Aztreonam (zone diameter ≥ 21 mm), 9 isolates (60%) harbored the blaIMP gene, and 6 isolates (40%) harbored the blaNDM gene (Tables 7 and 8).

#### MIC of ceftazidime/avibactam alone and in combination with aztreonam

**Fig. 4:**
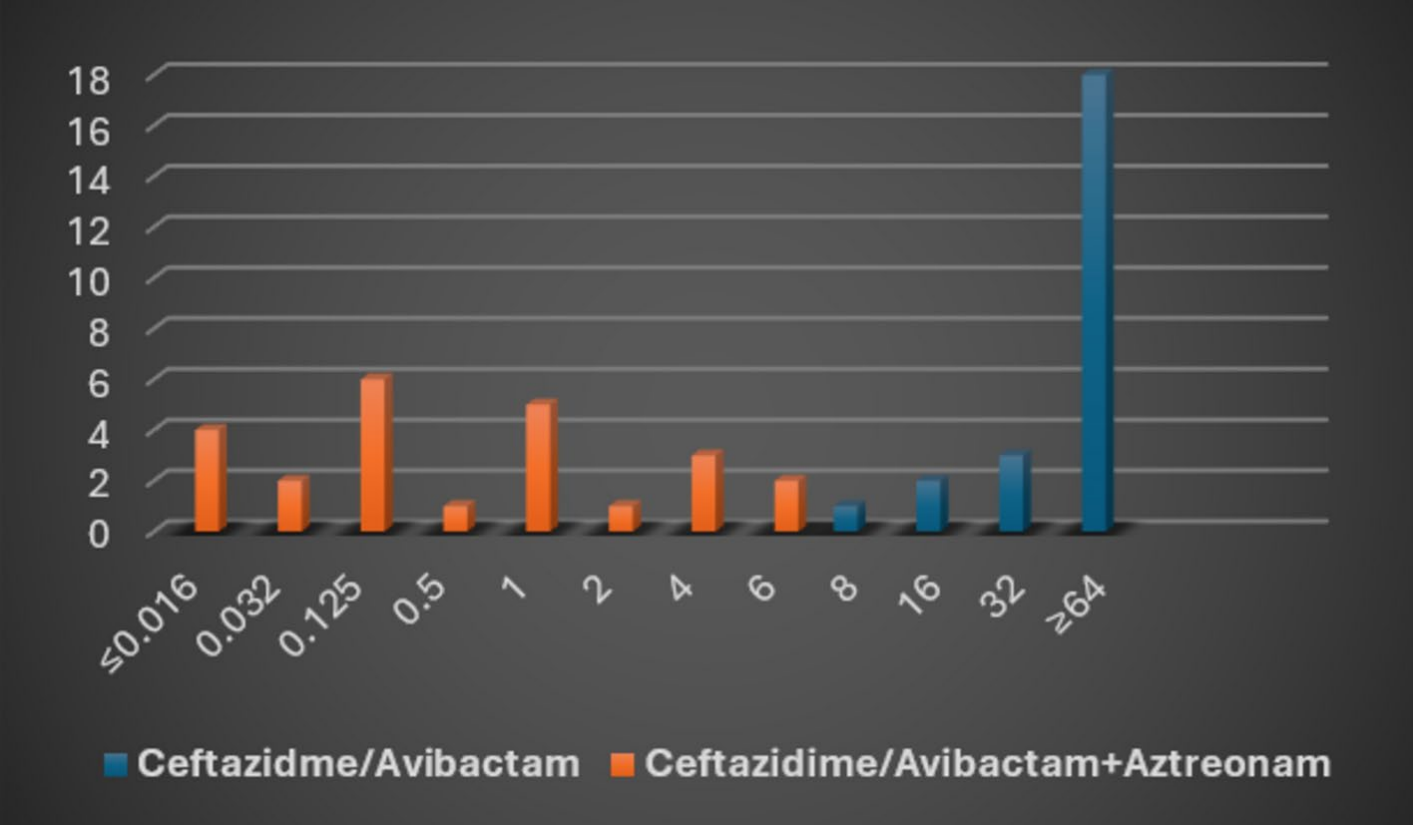
MIC of ceftazidime/avibactam alone and in combination with aztreonam by E-test strip.

**Fig. 5:**
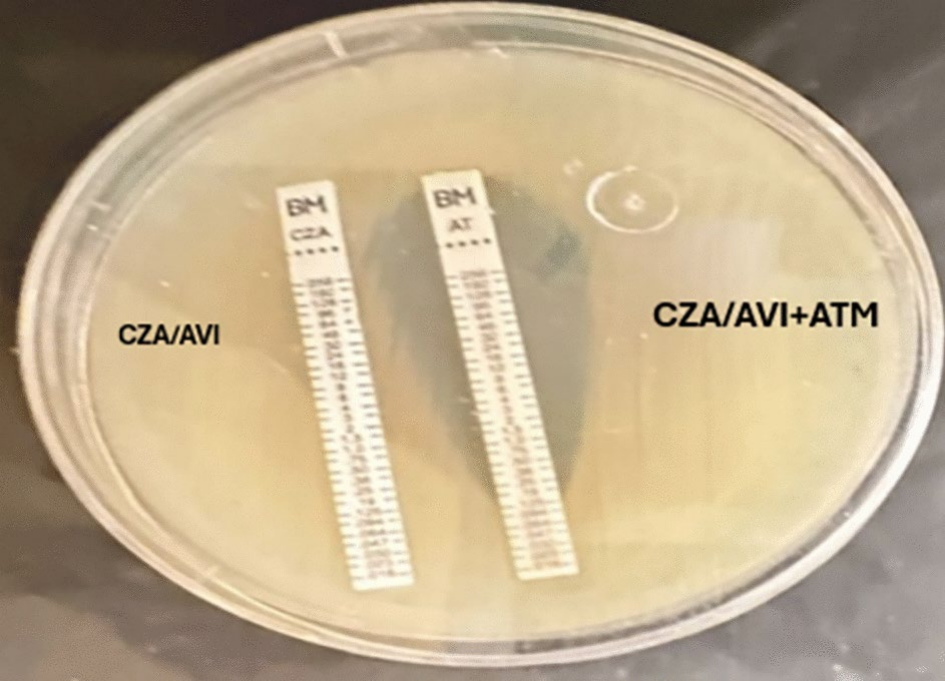
Antibiotic susceptibility by using E-test strips showing ceftazidime/avibactam strip alone and ceftazidime/avibactam combined with aztreonam.

When the minimal inhibitory concentration (MIC) antibiotic susceptibility test was done by E-test strips, it revealed MIC of ceftazidime/avibactam ranged from 8/4 to ≥ 64/4 µg/ml, while after doing the combination of ceftazidime/avibactam plus Aztreonam the MIC of all isolates ranged from ≤ 0.016/4 to 6/4 µg/ml (Figs. 4 and 5).

## Discussion

Carbapenem-resistant Klebsiella pneumoniae (CRKP) is a major cause of hospital-acquired infections, posing a significant threat to human health due to limited therapeutic options^9^. The combination of Ceftazidime/Avibactam (CAZ/AVI) and Aztreonam (ATM) has emerged as a crucial therapeutic option for MBL-producing CRKP, as it neutralizes class A, C, and some class D beta-lactamases while combating MBLs^20^. This study evaluated the in vitro efficacy of CAZ/AVI + ATM against MBL-producing CRKP isolates from Suez Canal University Hospital, Egypt.

The study was carried out on 365 clinical samples, a total of 65 K*. pneumoniae* isolates were recovered (17%). 60% (39/65) of the isolates were carbapenem-resistant *K. pneumoniae* (CRKP) as identified by either being meropenem- and/or imipenem-resistant. The age of the patients ranged from 16 to 75 years old, with a percentage of (61.5%) from male patients and (38.5%) from female patients (Table 2). The highest rate of CRKP isolation was from endotracheal aspirates (46.7%), while the lowest rate was from sputum and blood (10%) from the different ICUs (Table 2).

The CRKP prevalence has been reported worldwide. ICU hospitalization was found to be a significant risk factor for developing CRKP^21–24^. The disparities in prevalence rates may be related to changes in the hospital setting, the number of specimens examined, and the frequent use of carbapenems as empiric therapy in ICUs along with the antimicrobial stewardship program’s non-implementation. Studies from other hospitals in Egypt showed different prevalence rates of CRKP. It was 66%, 33.3%, 44.3%, and 14.2%, in Zagazig^21,22^, Mansoura^23^, Suez Canal^24^, and Al-Azhar^25^ University Hospitals respectively. The prevalence rate was 13.9% at the Egyptian National Cancer Institute-Cairo^26^. Comparably, other research revealed that the prevalence rate in New York and Greece was 20 and 40% respectively^27,28^. A study conducted in the USA revealed a higher result of 83%^29^.

The present study showed that the modified carbapenem inactivation method (mCIM) test showed that 35/39 (89.7%) CRKP isolates showed a zone diameter of 6–15 mm for meropenem disc and interpreted as carbapenemase positive, while 1/39 (2.6%) isolate showed a 17 mm zone diameter that was interpreted as carbapenemase intermediate and 3/39 (7.7%) isolates showed zone diameters of ≥ 19 mm and interpreted as carbapenemase negative (Table 3). The eCIM testing of the 35 carbapenemase-positive CRKP isolates showed that 24 isolates (68.6%) had an increase of zone diameter ≥ 5 mm after adding EDTA and they were considered Metallo-β-lactamase positive isolates (Table 4).

Previous studies have widely used the mCIM test for phenotypic carbapenemase detection. In Egypt, studies at Zagazig University hospitals reported 86% (49/57) and 89.1% (106/119) of CRKP isolates as carbapenemase-positive^21,22^, while Alexandria University Hospital found 70% (37/53) of isolates to be mCIM-positive, with 51% (19/37) confirmed as MBL-positive by eCIM^30^. In India, a study at a tertiary hospital revealed 96% (145/151) of CRKP isolates as mCIM-positive, with 98% (142/145) being eCIM-positive^31^. Similarly, studies in China reported 96.4% (402/417) and 88% (91/103) of isolates as mCIM-positive, with 26.4% (110/417) and 75% (68/91) being eCIM-positive, respectively^32,33^.

The modified carbapenem inactivation method (mCIM) effectively identifies carbapenemases but cannot differentiate between serine and metallo-β-lactamases (MBLs). To address this, the EDTA-modified carbapenem inactivation method (eCIM) was developed, enabling precise detection of MBLs. The combination of mCIM and eCIM tests distinguishes between serine carbapenemases (mCIM-positive, eCIM-negative) and MBLs (mCIM-positive, eCIM-positive)^34,35^. While mCIM is cost-effective, easy to perform, and requires no specialized equipment, its long incubation period (8 h to overnight) and inability to confirm carbapenemase class remains limitations^17^.

In the present study, by conventional PCR, molecular testing was done to detect the 3 MBL genes (*bla*IMP*, bla*NDM*, and bla*VIM) in the 24 Metallo-β-lactamase positive isolates. All 24 isolates (100%) harbored at least one of the MBL genes. A total of 22 isolates (91.7%) harbored *bla*IMP gene, 16 isolates (66.7%) harbored *bla*NDM and 2 isolates (8.3%) harbored *bla*VIM (Table 5, Fig. 2A-C).

**Table 5:** Distribution of isolates carrying MBL genes.

Gene	No. of positive isolates	%
*bla*NDM	16	66.7%
*bla*IMP	22	91.7%
*bla*VIM	2	8.3%

The CRKP isolates have genes that encode carbapenemase, such as *bla* genes, in addition to a range of genes that confer resistance to medications other than β-lactams, rendering all antibiotics ineffective. Carbapenemases are classified into two classes according to the composition of their active sites: “serine-carbapenemases” which have “Serine” in the active site making the class A and class D, and the second category “Metallo-β-lactamases (MBL)” which have an active site including a zinc atom making the class B. The most commonly reported genes in class B MBL-producing strains are *bla*NDM, *bla*IMP, and *bla*VIM, while the most commonly reported genes in class A and D are *bla*KPC and *bla*OXA, respectively^36^. Currently IMP-producing and NDM-producing CRKP are well recognized. Nevertheless, there haven’t been many reports of CRKP when *bla*IMP and *bla*NDM coexist^37–39^. Unlike our findings, several studies in Egypt revealed that the most prevalent gene among CRKP isolates was the *bla*NDM gene^21,40–42^. In Uganda, *bla*VIM was the most amplified carbapenemase gene, while *bla*NDM was the least amplified one^43^. Meanwhile, it was reported in China that *bla*KPC was the most prevailing gene in *K. pneumoniae*^44^. In Turkey, the *bla*OXA-48 gene was the most predominant followed by *bla*NDM and *bla*VIM genes respectively. *bla*KPC and *bla*IMP genes were not identified ^45,46^. In Saudia Arabia, the most common carbapenemases were the blaOXA-48 and blaNDM genes. No *bla*KPC or *bla*IMP genes were detected^47,48^.

In the present study, antibiotic susceptibility testing was done on the 39 K*. pneumoniae* isolates by the Kirby-Bauer disk diffusion method. All 39 CRKP isolates in the present study were multidrug-resistant (MDR). The highest resistance was for Ampicillin 39/39 (100%), Amoxicillin-clavulanate 37/39 (94.9%), Ceftazidime 32/39 (82.1%), and Cefotaxime 27/39 (69.2%) antibiotics. The most sensitive antibiotics were Cefepime 16/39 (41%) and Ciprofloxacin 14/39 (35.9%) (Table 6, Fig. 3).

**Table 6:** Antibiotic susceptibility pattern of the 39 CRKP isolates by Kirby-Bauer disk diffusion method.

Antibiotic	Susceptible		Intermediate		Resistant	
	No	%	No	%	No	%
Amoxicillin-clavulanate	0	0	2	5.1	37	94.9
Ampicillin	0	0	0	0	39	100
Cefotaxime	4	10.3	8	20.5	27	69.2
Ceftazidime	4	10.3	3	7.7	32	82.1
Cefepime	16	41	12	30.8	11	28.2
Ciprofloxacin	14	35.9	6	15.4	19	48.7
Levofloxacin	6	15.4	9	23.1	24	61.5
Gentamicin	8	20.5	11	28.2	20	51.3
Amikacin	7	17.9	9	23.1	23	58.9

Numerous investigations have also documented the noteworthy correlation between multidrug resistance and carbapenem resistance in K. pneumoniae isolates whether locally in Egypt^21–26^ or worldwide^32,49–51^. An alarming elevated prevalence of multidrug-resistant (MDR) “resistance to at least one agent in three or more antimicrobial classes”, extremely drug-resistant (XDR) “resistance to at least one agent in all but two or fewer antimicrobials”, and pan-drug-resistant (PDR) “resistance to all agents in all antimicrobial classes”. Since pan-drug-resistant isolates are essentially unaffected by antibiotics, higher rates of morbidity and mortality are to be predicted, which is concerning^52^.

*K. pneumoniae* resistance to carbapenems arises from complex mechanisms, including increased efflux pump activity, altered outer membrane permeability, and the production of carbapenemases, which hydrolyze β-lactams and may include extended-spectrum β-lactamases (ESBLs). According to CLSI surveillance data, 50–60% of carbapenem-resistant Enterobacterales remain susceptible to cefepime^53^, which may be used to treat these isolates despite carbapenemase production^54^. However, cefepime susceptibility should alert clinicians to potential treatment failure, as carbapenem-resistant isolates often exhibit multidrug-resistant (MDR) phenotypes and carry additional resistance factors. The expression of mobile genetic elements within carbapenemase genes further exacerbates resistance^55^, underscoring the urgent need for new antibiotics and synergistic combinations to combat these pathogens.

In our study, none of the isolates showed zone diameter ≥ 21 mm when testing Aztreonam disc susceptibility by the Kirby-Bauer method, while 2 isolates (8.3%) were ≥ 21 mm for Ceftazidime-Avibactam disc. However, after doing the combination method for Ceftazidime-Avibactam plus Aztreonam, synergy was observed in 15/24 (62.5%) of the resistant isolates, inhibition zone became ≥ 21 mm, indicating a shift from resistance to sensitivity. Among the 15 isolates that became sensitive to the combination of Ceftazidime-Avibactam + Aztreonam (zone diameter ≥ 21 mm), 9 isolates (60%) harbored the blaIMP gene, and 6 isolates (40%) harbored the blaNDM gene. None of the sensitive isolates carried the blaVIM gene, suggesting that the combination therapy is particularly effective against isolates carrying *bla*IMP and *bla*NDM genes (Tables 7 and 8). In addition, Ceftazidime-avibactam minimum inhibitory concentration (MIC) using E-test strips, ranged from 8/4 to ≥ 64/4 µg/ml (resistant) when tested. However, when ceftazidime-avibactam plus Aztreonam was combined, the MIC ranged from ≤ 0.016/4 to 6/4 µg/ml (sensitive) (Figs. 4 and 5).

**Table 7:** Antibiotic susceptibility testing of ceftazidime/avibactam, aztreonam, and ceftazidime/avibactam plus aztreonam for the 24 MBL isolates.

Isolate number		Ceftazidime/avibactam		Aztreonam		Ceftazidime/avibactam + aztreonam
No	MBL genes	Zone diameter (mm)	Susceptibility (≥ 21 S, ≤ 20R)	Zone diameter (mm)	Susceptibility (≥ 21S, 18-20I, ≤ 17R)	Zone diameter (mm)
1	*bla*IMP	13	R	6	R	21
2	*bla*NDM	15	R	10	R	21
3	*bla*IMP	19	R	19	I	27
4	*bla*IMP	15	R	6	R	23
5	*bla*NDM	6	R	6	R	11
6	*bla*IMP	13	R	6	R	22
7	*bla*IMP	20	R	6	R	11
8	*bla*NDM	6	R	6	R	15
9	*bla*IMP	6	S	6	R	21
10	*bla*NDM	17	R	6	R	22
11	*bla*IMP	15	R	6	R	17
12	*bla*IMP	17	R	6	R	21
13	*bla*NDM	21	S	17	R	24
14	*bla*IMP	12	R	6	R	14
15	*bla*NDM	14	R	9	R	21
16	*bla*IMP	18	R	18	I	26
17	*bla*NDM	16	R	8	R	21
18	*bla*IMP	7	R	6	R	23
19	*bla*NDM	14	R	8	R	17
20	*bla*IMP	21	S	6	R	10
21	*bla*VIM	7	R	6	R	16
22	*bla*VIM	7	R	6	R	17
23	*bla*IMP	18	R	6	R	22
24	*bla*NDM	14	R	6	R	22
Total		13.79 ± 4.93^bc^		7.96 ± 4.04^ac^		19.33 ± 4.63^ab^

ANOVA test used. Tukey Post Hoc test was used.

^a^Statistically significant with the ceftazidime-avibactam group.

^b^Statistically significant with the aztreonam group.

^c^Statistically significant with the ceftazidime/avibactam + aztreonam group.

*F-*value 37.514.

*P-*value < 0.001*

**Table 8:** Number of isolates showing inhibition zone diameter ≥ 21 mm when testing aztreonam, ceftazidime/avibactam, alone and combined ceftazidime/avibactam plus aztreonam.

Ceftazidime/avibactam (≥ 21 mm)		Aztreonam (≥ 21 mm)		Ceftazidime/avibactam plus aztreonam (≥ 21 mm)		*X*^*2*^	*P-*value
No. of isolates	%	No. of isolates	%	No. of isolates	%		
2	8.3	0	0	15	62.5	30.648	< 0.001*

*Fisher exact test used.

Previous studies revealed promising results of the combination of CAZ/AVI and ATM to treat carbapenem-resistant Enterobacterales. Synergy was observed between CAZ/AVI and ATM disc in 33/35 (94.28%)^56^ and 60/60 (100%)^57^ of the carbapenem-resistant *Klebsiella species* isolates. Several recent investigations have also shown, through the use of various testing methodologies, the synergy between CAZ/AVI and ATM in MDR, XDR, and PDR *Klebsiella species* isolates^15,58–61^.

Ceftazidime/Avibactam, a third-generation cephalosporin coupled with a β-lactamase inhibitor, is a new and efficacious therapeutic alternative that exhibits broad-spectrum efficacy against serine β-lactamases but is degraded by Metallo-β-lactamases. Aztreonam, on the other hand, is vulnerable to hydrolysis by serine β-lactamases but stable in the presence of Metallo-β-lactamases. Thus, Ceftazidime/Avibactam + Aztreonam is a useful combination to treat infections caused by Gram-negative bacteria that produce serine β-lactamases and/or Metallo-beta-lactamases^14^.

This study has several limitations. First, the detection of resistance genes (*blaNDM*, *blaIMP*, and *blaVIM*) was performed using conventional PCR, which confirms their presence but does not provide detailed sequence information or identify specific variants. Future studies should include sequencing to characterize these genes further and explore potential mutations or variants. Second, the study was conducted at a single center, which may limit the generalizability of the findings. Finally, the sample size, though sufficient for preliminary analysis, could be expanded in future work to strengthen the statistical power of the results.

## Conclusion

The novel combination of Ceftazidime/Avibactam (CAZ/AVI) and Aztreonam (ATM) demonstrates significant in vitro efficacy against Metallo-β-lactamase-producing *Klebsiella pneumoniae* strains. This combination effectively overcomes resistance mediated by MBLs and serine β-lactamases, offering a promising therapeutic option for treating highly resistant CRKP infections. Nevertheless, more in vivo studies are still required to validate these findings and explore the clinical applicability of this combination therapy.

## Data Availability

All data generated or analyzed during this study are included in this published article.
